# Experiences from the Philippine grassroots: impact of strengthening primary care systems on health worker satisfaction and intention to stay

**DOI:** 10.1186/s12913-022-08799-1

**Published:** 2023-02-04

**Authors:** Regine Ynez H. De Mesa, Jose Rafael A. Marfori, Noleen Marie C. Fabian, Romelei Camiling-Alfonso, Mark Anthony U. Javelosa, Nannette Bernal-Sundiang, Leonila F. Dans, Ysabela T. Calderon, Jayson A. Celeste, Josephine T. Sanchez, Mia P. Rey, Cara Lois T. Galingana, Ramon Pedro P. Paterno, Jesusa T. Catabui, Johanna Faye E. Lopez, Maria Rhodora N. Aquino, Antonio Miguel L. Dans

**Affiliations:** 1 University of the Philippines Diliman, Quezon City, Philippines; 2 University of the Philippines Manila, Manila, Philippines

**Keywords:** Primary health care, Health workforce, Healthcare disparities, Job satisfaction, Intervention study

## Abstract

**Background:**

Inequities in health access and outcomes persist in low- and middle-income countries. While strengthening primary care is integral in improving patient outcomes, primary care networks remain undervalued, underfunded, and underdeveloped in many LMICs such as the Philippines. This paper underscores the value of strengthening primary care system interventions in LMICs by examining their impact on job satisfaction and intention to stay among healthcare workers in the Philippines.

**Methods:**

This study was conducted in urban, rural, and remote settings in the Philippines. A total of 36 urban, 54 rural, and 117 remote healthcare workers participated in the study. Respondents comprised all family physicians, nurses, midwives, community health workers, and staff involved in the delivery of primary care services from the sites. A questionnaire examining job satisfaction (motivators) and dissatisfaction (hygiene) factors was distributed to healthcare workers before and after system interventions were introduced across sites. Interventions included the introduction of performance-based incentives, the adoption of electronic health records, and the enhancement of diagnostic and pharmaceutical capabilities over a 1-year period. A Wilcoxon signed-rank test and a McNemar’s chi-square test were then conducted to compare pre- and post-intervention experiences for each setting.

**Results:**

Among the factors examined, results revealed a significant improvement in perceived compensation fairness among urban (*p* = 0.001) and rural (*p* = 0.016) providers. The rural workforce also reported a significant improvement in medicine access (*p* = 0.012) post-intervention. Job motivation and turnover intention were sustained in urban and rural settings between periods. Despite the interventions introduced, a decline in perceptions towards supply accessibility, job security, and most items classified as job motivators was reported among remote providers. Paralleling this decline, remote primary care providers with the intent to stay dropped from 93% at baseline to 75% at endline (*p* < 0.001).

**Conclusion:**

The impact of strengthening primary care on health workforce satisfaction and turnover intention varied across urban, rural, and remote settings. While select interventions such as improving compensation were promising for better-supported settings, the immediate impact of these interventions was inadequate in offsetting the infrastructural and staffing gaps experienced in disadvantaged areas. Unless these problems are comprehensively addressed, satisfaction will remain low, workforce attrition will persist as a problem, and marginalized communities will be underserved.

**Supplementary Information:**

The online version contains supplementary material available at 10.1186/s12913-022-08799-1.

## Introduction

### Background of the study

The passage of the Universal Health Care Law in 2019 marked the Philippines’ commitment to achieve equitable health coverage for all, an endeavor shared by countries worldwide [1]. However, even if primary care is acknowledged as an essential element to achieving universal health coverage [1–3] and as a mechanism for improved health equity [4], the lack of health system readiness remains an issue. In the Philippines, as in many low- and middle-income countries (LMICs) [5, 6], primary care networks remain undervalued, underfunded, and ultimately underdeveloped [7]. This has contributed to compromised health outcomes such as significantly shorter life expectancies [8] and higher child mortality rates among the country’s poorest quintiles [9]. National data reveals that 6 out of 10 Filipino deaths were medically unattended—with only the capital region of Metro Manila exhibiting higher attended than unattended deaths [10]. The World Health Organization sets the ideal skilled health worker (i.e., physicians, nurses, and midwives) to population ratio at 4.45:1000 [11]. However, human resources for health (HRH) deficits of at least 60,000 doctors, 121,000 nurses, and 109,000 midwives were reported among Philippine public facilities alone [12]. This bears significance as over 83% of outpatient visits from the two poorest wealth quintiles were made to government-funded community health stations [13].

Largely driven by workforce maldistribution and system fragmentation, inequities in health have persisted in the absence of a well-supported primary care network [14]. Disparities in health access have likewise had a disproportionate impact on low-income families [13] and geographically disadvantaged regions [15]. In rural and remote areas, health stations are often gravely understaffed, lacking supplies of basic drugs, and left without the regular supervision of an attending physician [16]. Despite severe HRH shortages, the Philippines remains a leading exporter of health professionals with nearly 85% of locally trained nurses deployed overseas [17]. The country’s economic reliance on the mass exodus of its workforce without comprehensively addressing the steep decline in HRH retention has adversely impacted health service delivery in underserved communities [18]. Thus, improving the retention of HRH is at the cornerstone of operationalizing primary care systems in LMICs like the Philippines.

Literature examining emigration patterns forward the substantial impact of job satisfaction on turnover intention. Locke broadly defines job satisfaction as “a pleasurable or positive emotional state resulting from the appraisal of one’s job or job experiences” [19]. A myriad of job attributes, such as workload, training opportunities, compensation, and enabling environments, influence job satisfaction [20]. Herzberg’s two-factor theory distinguishes between attributes that induce satisfaction from those that lead to dissatisfaction. These attributes are categorized as a) motivators and b) hygiene factors [21]. Motivators such as workplace morale and job involvement were linked to employee satisfaction, whereas hygiene factors such as compensation and job security were associated with dissatisfaction if not aptly addressed [22]. While Herzberg’s theory suggests that the absence of motivators may not necessarily lead to attrition, environments that only support good workplace hygiene can result in retaining an unsatisfied workforce [23]. Exploring the impact of strengthened primary care on HCW satisfaction has widespread implications for health outcomes in LMICs. Sustained contentment towards various workplace attributes enables HCWs to direct optimal focus towards patient care. With low HCW retention directly resulting in poor outcomes [24], HCW satisfaction proves integral for mitigating the effects of workforce maldistribution in LMICs.

### Study objectives

Adopting Herzberg’s two-factor framework for analyzing job satisfaction, the objectives of this study are: 1) to evaluate the impact of strengthening urban, rural, and remote primary care system interventions on HCW satisfaction; and 2) to compare turnover intention among HCWs before and after the intervention period.

## Methodology

### Study design

A pretest-posttest design was used to assess HCW job satisfaction and turnover intention across urban, rural, and remote settings in the Philippines. This entailed data collection of satisfaction measures through a single pre-test, followed by an intervention, and then a collection of post-test data on the same measure. The present study was conducted as part of the Philippine Primary Care Studies (PPCS) program, a longitudinal and multi-sited research series aimed at strengthening primary care systems through patient-centered interventions. In 2016, PPCS piloted its urban program to model comprehensive primary care, which provided free access to outpatient services, laboratory and diagnostic procedures, and medicines to eligible patients. This model and its package of interventions were extended to the study’s rural and remote sites in 2019. In delivering these interventions, HCWs were supported through enhanced capacity-building, the development of electronic health records (EHR), and the introduction of performance-based financial incentives. The impact of these interventions was then assessed, with HCW job satisfaction being one of the eight health system outcome measures outlined in the PPCS primary care model [25].

### Instrumentation

A pre-validated Stayers questionnaire [26] initially used to measure job satisfaction and turnover intention among remote primary care physicians [27] was adapted for this study. The adapted instrument comprised a Likert-type section to measure satisfaction/dissatisfaction and a multiple-choice assessment to measure turnover intention. Following Herzberg’s two-factor framework, Likert items were classified as: 1) motivator factors; and 2) hygiene factors (see Table 1).

**Table 1:** List of Likert scale items by Herzberg’s two-factor classification

Motivator Factors (α = 0.8)		Hygiene Factors (α = 0.7)	
Q1	Job satisfaction	Q6	Workload
Q2	Workplace morale	Q7	Access to supplies
Q3	Recommendability	Q8	Access to equipment
Q4	Enjoyment	Q9	Access to medicines
Q5	Job involvement	Q10	Job security
		Q11	Compensation fairness

Since the original instrument was used to measure physician satisfaction in a remote setting, several sections of the original questionnaire did not apply to the practice types and settings examined in this study. As such, only 18 of the original 77 items were maintained (see Appendix A) to enhance questionnaire adaptability. Overall consistency for the tool used in this study proved reliable [28] with a Cronbach’s α score of 0.8.

### Sampling and survey distribution

This study was conducted across three pilot sites, namely: a) an urban site—the University of the Philippines Health Service Diliman in Metro Manila; b) the rural municipality of Samal, Bataan; and c) the remote municipality of Bulusan, Sorsogon. A census of all HCWs from the urban, rural, and remote sites was obtained—totaling 36, 54, and 117 respondents respectively. Self-administered questionnaires were distributed to the respondents in September 2016 for the baseline period and again in December 2017 for the endline assessment at the urban site. Baseline surveys for rural and remote sites were distributed during the study preparation phase in April 2019 and assessed after the one-year implementation in June 2020 through the endline survey. Verbal and written consent from each respondent was obtained before survey distribution.

### Data analysis

Data gathered were encoded in Microsoft Excel and were analyzed using Stata version 12.0 and R version 3.5.0. Demographics were expressed through percentage comparisons for categorical variables, whereas mean scores were used to compare continuous data. Likert responses were scored as 1 = Strongly Disagree (SD); 2 = Disagree (D); 3 = Neutral (N); 4 = Agree (A); 5 = Strongly Agree (SA). Upon analysis, Likert-type responses were examined per item and as dichotomized responses (i.e., generally dissatisfied for scores 1–3 and generally satisfied for scores 4–5) [29–31]. Multiple-choice items on intent to stay were encoded as a binary before analysis. HCWs intending to leave were segregated from HCWs intending to stay in their jobs indefinitely. To determine the significance between baseline and endline scores, hypothesis testing was conducted using the Wilcoxon signed-rank test for ordinal data and McNemar’s chi-square test for dichotomous data. Hodges-Lehmann point estimates reflecting the direction of change in Likert satisfaction scores between periods were reported along with their 95% confidence intervals (see Appendix B). *P*-values of < 0.05 were considered statistically significant for this study. Ethical approval for this study was obtained from the University of the Philippines Manila Research Ethics Board (UPMREB – 2015-489-01) and the Philippines’ Department of Health Single Joint Research Ethics Board (SJREB – 2029-55). Ethics approval was annually renewed for all study sites. Furthermore, verbal and written informed consent was obtained from all health workers who have participated in this study.

## Results

### Demographic profile

Majority of our respondents were female. Our participants were HCWs from the sites and included family physicians, nurses, midwives, community health workers, and staff. The urban site had the most number of physicians while the rural and remote sites being serviced by community health workers (CHWs or locally referred to as *barangay* health workers) who bridge the gap between health systems and localities [25]. The distribution of HCWs, particularly the doctor to patient ratio, reflects the disparities across communities. The average length of stay in years was also highest in the urban site (14 years) and lowest in the remote site (11 years). Most HCWs from the urban (86%) and remote (73%) sites reported no previous work experience apart from the job they occupied during the survey period (Table 2).

**Table 2:** Demographic profile of survey respondents

Variables	Urban (*N* = 36)		Rural (*N* = 54)		Remote (*N* = 117)	
	n	%	n	%	n	%
**Sex**						
Female	24	67%	51	94%	114	97%
Male	12	33%	3	6%	3	3%
**Job description**						
Administrative aide	6	17%	2	4%	5	4%
Community health worker	0	0.0%	19	35%	99	85%
Medical doctor	9	25%	1	2%	0	0%
Medical technologist	4	11%	1	2%	1	1%
Midwife	5	14%	16	30%	8	7%
Nurse	8	22%	10	19%	4	3%
Pharmacist	4	11%	0	0%	0	0%
Other	0	0.0%	5	9%	0	0%
**Job Experience**						
No previous job experience	31	86%	26	48%	85	73%
**Job Tenure**						
Mean length of stay in years	14	–	12	–	11	–

### Comparison of health worker satisfaction across sites

The baseline proportion of generally satisfied HCWs was relatively low at the urban site compared to rural and remote responses towards job motivators. While almost all urban HCWs felt secure with their current jobs (92%), far less perceived their work as enjoyable (63%) or felt positively towards their workplace morale (72%) at baseline. In contrast to urban data, the majority of rural and remote HCWs (> 85%) were generally more motivated despite experiencing moderate to low workplace hygiene at the start of the study period. Among hygiene factors, over half of the workforce across all sites felt undercompensated during the baseline period. The baseline percentage of HCWs satisfied with their job hygiene was lowest at the remote site, with less than 30% of remote HCWs expressing sufficient access to medical equipment (Table 3).

**Table 3:** McNemar’s chi-square comparison of generally satisfied HCWs from the baseline and endline periods

	Urban (*N* = 36)			Rural (*N* = 54)			Remote (*N* = 117)		
Variables	Baseline	Endline	*p*-value	Baseline	Endline	*p*-value	Baseline	Endline	*p*-value
**Motivation Factors**									
Job satisfaction	80.0%	88.6%	0.317	90.7%	88.9%	0.739	86.2%	85.3%	0.808
Workplace morale	72.2%	75.0%	0.782	96.2%	90.6%	0.257	88.9%	77.8%	0.016*
Recommendability	91.4%	91.4%	1.000	98.1%	90.6%	0.102	94.0%	89.7%	0.225
Enjoyment	62.9%	71.4%	0.366	94.3%	94.3%	1.000	92.2%	82.6%	0.028*
Job involvement	91.7%	86.1%	0.414	96.2%	98.1%	0.564	96.5%	94.0%	0.366
**Hygiene Factors**									
Workload	91.7%	86.1%	0.414	90.7%	92.6%	0.739	80.2%	78.4%	0.746
Access to supplies	78.8%	69.7%	0.405	80.4%	72.5%	0.317	60.3%	52.6%	0.199
Access to equipment	54.8%	71.0%	0.197	58.0%	32.0%	0.007*	29.8%	41.2%	0.058
Access to medicines	54.2%	37.1%	0.109	48.1%	73.1%	0.009*	51.3%	56.4%	0.366
Job security	91.7%	88.9%	0.705	74.1%	64.8%	0.275	65.5%	58.4%	0.228
Compensation	40.0%	71.4%	0.008*	45.3%	56.6%	0.221	40.0%	33.0%	0.238

* p < 0.05; statistically significant difference in the proportion of generally satisfied responses

We found a marked increase in the endline proportion of generally satisfied HCWs towards perceived compensation fairness at the urban and rural facilities and access to medicines at the rural site. However, significantly fewer rural HCWs felt satisfied with the accessibility of equipment during the endline period. The endline proportion of satisfied urban and rural HCWs remained constant towards motivation factors. However, there was a decrease in satisfaction on workplace morale and enjoyment towards HCWs working in the remote community.

We also found two notable improvements in scores, specifically in: a) perceived compensation fairness among urban (*p* = 0.001) and rural HCWs (*p* = 0.016), and b) perceived sufficiency in medicine supply among rural HCWs (*p* = 0.012). Point estimates on the change in scores for both sites suggest that median satisfaction on perceived compensation fairness increased by a full rank (Table 4). For the urban and rural cohorts, median satisfaction scores increased from being neither satisfied nor dissatisfied [3] at baseline to being satisfied [4] at the end of the study (see Appendix B).

**Table 4:** Wilcoxon signed-rank test comparison of median satisfaction scores between baseline and endline periods

	Urban (*N* = 36) Median (Range)			Rural (*N* = 54) Median (Range)		Remote (*N* = 117) Median (Range)			
Domain	Baseline	Endline	*p*-value	Baseline	Endline	*p*-value	Baseline	Endline	*p*-value
**Motivation Factors**									
Job satisfaction	4 (3–5)	4 (2–5)	0.292	5 (3–5)	5 (3–5)	0.545	5 (2–5)	4 (1–5)	0.056
Workplace morale	4 (1–5)	4 (1–5)	0.968	5 (3–5)	5 (3–5)	0.722	5 (2–5)	4 (2–5)	< 0.001**
Recommendability	4 (3–5)	4 (3–5)	0.835	5 (3–5)	5 (3–5)	0.375	5 (2–5)	4 (2–5)	0.011**
Enjoyment	4 (3–5)	4 (2–5)	0.433	5 (3–5)	5 (3–5)	0.585	5 (2–5)	4 (2–5)	0.002**
Job involvement	4 (3–5)	4 (3–5)	0.528	5 (3–5)	5 (3–5)	0.349	5 (2–5)	5 (2–5)	0.060
**Hygiene Factors**									
Workload	4 (2–5)	4 (2–5)	0.234	4 (3–5)	4 (1–5)	0.741	4 (2–5)	4 (2–5)	0.663
Access to supplies	4 (3–5)	4 (2–5)	0.064	4 (2–5)	4 (2–5)	0.702	4 (2–5)	4 (1–5)	0.025**
Access to equipment	4 (2–5)	4 (2–5)	0.217	4 (1–5)	3 (1–5)	0.091	3 (2–5)	3 (1–5)	0.198
Access to medicines	4 (2–5)	3 (2–5)	0.528	3 (1–5)	4 (2–5)	0.012*	4 (2–5)	4 (2–5)	0.576
Perceived job security	4 (3–5)	4 (2–5)	0.686	4 (1–5)	4 (3–5)	0.904	4 (2–5)	4 (2–5)	0.020**
Compensation fairness	3 (1–5)	4 (3–5)	0.001*	3 (1–5)	4 (1–5)	0.016*	3 (2–5)	3 (1–5)	0.384

1 *P* < 0.05; statistically significant increase between baseline and endline ranks

** *P* < 0.05; statistically significant decrease between baseline and endline ranks

When remote data were analyzed, significantly lower satisfaction scores were reported for both motivator and hygiene factors. Satisfaction towards motivating factors was significantly lower at the end of the study—with workplace morale exhibiting the steepest decline (*p* < 0.001). Remote HCWs also reported lower satisfaction scores towards hygiene factors like supply accessibility and job security post-intervention. In contrast to urban andrural responses, no statistically significant changes were noted in perceived compensation fairness at the remote site.

### Comparison of intention to stay across sites

Intention to stay did not change in the urban site after the primary care system interventions (*p* = 1.000*)*. More HCWs in the rural site indicated intention to stay (baseline: 75% vs. endline: 89%; *p* = 0.090) while fewer HCWs practicing in the remote site intended to stay post-intervention (baseline: 93% vs. endline: 76%; *p* < 0.001) (Table 5).

**Table 5:** McNemar’s chi-square test results on intent-to-stay across sites

Site	Baseline		Endline		*P-value*
	n/N	%	n/N	%	
**Urban**	26/35	74%	26/35	74%	1.000
**Rural**	39/52	75%	46/52	89%	0.090
**Remote**	100/107	93%	81/107	76%	< 0.001*

* *P* < 0.05; statistically significant difference in the proportion of generally satisfied responses

## Discussion

Health sector performance hinges on a competent, motivated, and well-supported workforce. If performance gains are to be realized when transitioning from vertical disease-based health programs to integrated primary care systems, HCW satisfaction must be considered as a desired outcome measure. Technical training and enhanced incentives are necessary for improving HCW satisfaction [32]. However, the existing curricula of health-related professions in the Philippines have limited content and training on primary care. An appraisal conducted on HCW job motivation underscores a systemic approach in improving satisfaction scores and workforce retention [33]. According to existing literature, insufficient performance incentives and compensation have resulted in poor health outcomes and HCW maldistribution across challenging environments such as the Philippines [34–37]. Non-financial incentives also play a role in attracting physicians to practice in rural health systems, which includes supervision and being near to their families. To address maldistribution, this study initiated several interventions to encourage system integration and HCW capacity-building [38]. Primary care training workshops and access to UpToDate were provided to HCWs throughout the study period. Additional pharmacies and laboratories were incorporated into existing networks in the rural and remote sites to expand drug supply and services. A unified EHR system was also introduced to all sites to ease patient intake, diagnosis, referral, and monitoring.

In the study’s rural and remote sites, clinical care is delivered across a multitude of facilities. These range from central health units that house a limited number of physicians, to smaller community health stations that primarily operate through the services rendered by nurses, midwives, and CHWs. The introduction of the EHR enhanced system integration across these facilities through a unified patient database. In effect, the EHR enabled previously underutilized community health stations to refer patients to the central health unit and to likewise produce laboratory requests or prescriptions with the remote approval of the patient’s attending primary care physician. Rural HCWs were less dissatisfied with their ability to prescribe medical drugs post-intervention. As supported by post-intervention studies conducted in rural terrains, this likely resulted from the expansion of these services alongside the remote referral/approval capabilities provided by the EHR [39, 40]. The central health unit of the rural site experienced the highest number of consultations year-round. As such, the referral/approval capabilities aided in distributing patients across the network of available community health stations. While most rural satisfaction scores have remained consistent, majority of rural HCWs (> 90%) were already highly satisfied with all motivational factors during the baseline period. Considerable institutional support and tight integration pre-intervention may have contributed to the high confidence level demonstrated by rural HCWs at baseline [41]. Their overall satisfaction was mirrored in their greater intention to stay after the implementation of primary care system interventions.

Dissatisfaction towards perceived compensation fairness was consistently high pre-intervention. To address possible gaps in remuneration, performance-based financial incentives were provided to all primary care providers across the three sites during the intervention period. These incentives were calculated based on completed consultations by the involved HCWs per consult. When a patient is initially assessed by a nurse and referred to an attending physician, both HCWs would merit financial incentives in the implemented payment scheme. As indicated in research evaluating the impact of HCWs income, adequate wage provisions are vital to system-incentivized performance improvements [42, 43] and coordinated care among HCWs within the primary care network [25]. The results of this study reveal that perceptions towards compensation fairness significantly improved among urban and rural HCWs post-intervention. This may largely be due to the provision of the aforementioned incentives as wages and other fringe benefits across all sites remained the same.

Job hygiene at the remote site showed a conservative decline. Remote HCWs were more dissatisfied with supply accessibility and job security post-intervention. Although urban and rural job hygiene improved with the introduction of financial incentives, the remote site reported no significant difference in HCW perceptions towards perceived compensation fairness post-intervention. A slight decline in the level of satisfaction and the proportion of generally satisfied HCWs were also noted towards several motivation factors. Four underlying contexts can be examined to qualify these results: 1) delayed incentivization [36]; 2) HCW maldistribution [42]; 3) weak infrastructure [44]; and 4) the impact of COVID-19 [45]. Irregular payments and delayed remuneration contribute to HCW dissatisfaction and ultimately poor retention [46]. Resulting from administrative delays in the disbursement of additional financial incentives, most remote HCWs received these incentives several months after their services were rendered. This may have significantly mitigated the intended positive impact of incentivization. Although delays in incentive payouts occurred in other sites, the impact of delayed remuneration may have been more difficult to ignore in the remote site given the abundance of other challenges shouldered by its workforce.

Apart from administrative challenges, the demographic composition of remote-based staff likely had some impact on the reported dissatisfaction towards several hygiene factors. CHWs comprised the vast majority of the remote-based workforce surveyed in this study. CHWs are part-time volunteer workers, rendering them ineligible for receiving a regular wage, unlike other primary care providers. Non-urban CHWs typically receive a marginal monthly allowance of Php 1150 (estimated at $24.00 per month) alongside other benefits such as free groceries or medical care depending on the local government unit [47]. While intrinsic job factors such as perceived social prestige and acquired technical skills have been shown to be critical motivators for CHWs in existing literature [47], heightened dissatisfaction towards the inadequacy of job hygiene factors relative to the work expected may increase turnover intention as Herzberg’s theory and the findings of this study present.

The sporadic distribution of HCWs, particularly physicians, in remote areas proves potentially hazardous for providers—threatening to overload both staff and infrastructure. Expanding primary care providers’ responsibilities to include public health service delivery may cause low job satisfaction due to inadequate work autonomy and high dissatisfaction due to income mismatch [48]. HCWs are expected to deliver quality clinical services to individual patients while assuming population health roles for specific health programs (i.e., vaccination, sanitation). Despite the range of tasks HCWs are expected to fulfill, infrastructural gaps in the remote site vastly surpass those of other sites. Intermittent internet connectivity, unreliable transportation, poor maintenance of select health stations, and frequent electrical outages are additional challenges to an already understaffed workforce. ﻿These challenges potentially diminish health outcomes, rendering clinical efforts futile or frustrating, and may reinforce low regard for the primary care system—amongst providers and patients [44, 49]. ﻿With infrastructural lacunae and the regular onslaught of natural disasters in this Pacific-facing site, seemingly minor inconveniences have resulted in adverse delays. This is evident in hours of back-encoding patient data, longer patient queues, difficulties in servicing remote communities, and challenges in referring patients throughout the primary care network.

Enhanced retention necessitates providing basic resources required for the job—including improved infrastructure, a unified EHR, supply accessibility, and fair compensation. Furthermore, experiences from the remote site suggest that financial incentives prove more effective once other infrastructural hurdles have already been addressed. System interventions must indeed provide enabling environments to prevent dissatisfaction and reduce workforce attrition. However, as Herzberg’s theory posits, job satisfaction is primarily achieved with a motivated workforce. In the urban site, most HCWs were not dissatisfied with hygiene factors such as workload and overall job security. However, satisfaction with motivational factors was still lower compared to rural and remote scores. Despite being in a well-supported job environment that retained its workforce the longest compared to other sites, urban data shows that good job hygiene alone does not ascertain HCW satisfaction. Providing non-monetary incentives such as training opportunities, pathways for career advancement, and involvement in clinical decision-making proves foremost essential in improving job satisfaction.

### Scope and limitations

This study employed a diachronic approach in evaluating HCW satisfaction across three sites, with varying baseline and endline periods per site due to funding and infrastructural constraints. The endline responses from the rural and remote sites were obtained shortly after the onset of the COVID-19 pandemic. As such, the shifting social and economic climate may have affected responses at the time of the survey. Other factors such as survivor bias may have had some impact on the reported results. Only respondents with matched scores (i.e., HCWs present in both baseline and endline periods) were included for analysis. Other factors influencing satisfaction were not controlled. As such, the magnitude of each factor and its corresponding effect on satisfaction and intent to stay was outside the scope of the present study. Attempts to further contextualize satisfaction scores have been undertaken to grasp a holistic understanding of HCW experience. These were done through informal interviews with HCWs, and long-term participant observation of field teams deployed to each site. However, we were unable to measure the role of corruption in this study and we suggest that future studies collect data on this to better qualify and quantify its effect. With these limitations outlined, this research places greater focus on the possible impact of specific interventions undertaken in strengthening primary care networks in each area.

## Conclusion

This study presents the observed impact of strengthening urban, rural, and remote primary care system interventions on primary care providers. Using Herzberg’s two-factor classification, overall job satisfaction and turnover intention were examined through motivational and hygiene factors experienced in each site before and after the implementation of study interventions. Perceptions towards job hygiene factors improved post-intervention at urban and rural sites—likely because of performance-based financial incentives provided to all HCWs during the study. Alongside the provision of monetary incentives, the expansion of service delivery networks to include additional pharmacies in the rural site showed a positive impact among HCWs in their regard for medical supply.

Despite attempts to strengthen the existing primary care system and potentially exacerbated by the effects of the COVID-19 pandemic, infrastructural deficits have contributed to lower motivation and higher dissatisfaction among remote HCWs during the endline period. Reducing dissatisfaction by addressing hygiene factors at the workplace proves vital in retaining HCWs in remote and disadvantaged areas. This may be done by providing adequate remuneration and ensuring work environments support the demands of person-centered integrated care. However, targeting system interventions aimed at improving motivational factors may render beneficial in retaining a satisfied workforce in the long term. Strengthening primary care systems must, therefore, consider interventions that address motivational and job hygiene needs to improve healthcare worker satisfaction and intention to stay. This includes addressing HCW needs, strengthening infrastructural support, and enhancing primary care training across all HCW cadres. In doing so, patient-centered primary care can ultimately be better sustained by the very workforce it is founded upon.

## Supplementary Information

**Additional file 1.**

## Data Availability

The datasets generated and/or analyzed during the current study are not publicly available to uphold participant privacy. Datasets are available from the corresponding author on reasonable request.

## Abbreviations

LMIC: low- and middle-income country
HRH: human resources for health
HCW: healthcare worker
PPCS: Philippine Primary Care Studies
SD: strongly disagree
D: disagree
N: neither agree nor disagree
A: agree
SA: strongly agree
CHWs: community health worker
EHR: electronic health records
