# Supplementary material for: Experiences from the Philippine grassroots: impact of strengthening primary care systems on health worker satisfaction and intention to stay
Source: BMC Health Serv Res. 2023 Feb 4;23:117. doi: 10.1186/s12913-022-08799-1 (PMC9898850; doi:10.1186/s12913-022-08799-1)
Supplement: Supplementary file 1 — Additional file 1. [file 12913_2022_8799_MOESM1_ESM.docx]

## Appendix A

Health Worker Satisfaction and Retention Questionnaire

| We would like to evaluate your general satisfaction with your present work. Please write or encircle your answers to each question. Since this is part of an ongoing study being conducted by Philippine Primary Care Studies, your responses will be used as data for analysis. Results from this study may be used for future projections and studies. Please be assured that your responses will be kept strictly confidential. No personal identification will be retrieved or traced from this questionnaire. |
| --- |
| **Name (LAST NAME, First Name, Middle Name):** |
| **Age:** |

**Section I:** **Background Information**

Enter or check your answers to the questions below.

| **#** | **Questions** | **Enter or check your answers.** |
| --- | --- | --- |
| 1 | Sex Assigned at Birth | ☐ Male  ☐ Female |
| 2 | What is your profession? | ☐ Administrative Aide / Records / Billing  ☐ Medical Doctor  ☐ Medical Technologist  ☐ Nurse  ☐ Pharmacist  ☐ Radiologist  ☐ Dentist  ☐ Dental Assistant  ☐ Other: ______ |
| 3 | How long have you worked with this organization (e.g. UP Diliman/Municipality Health Office)? | _____ years  _____months |
| 4 | How long have you been at this facility (e.g. UPHS / RHU/ BHS)? | _____ years  ____months |
| 5 | Is this your first job since receiving your most recent qualification? | ☐ Yes  ☐ No |

**Section II: Job Satisfaction and Morale**

The following questions refer to your job satisfaction and morale where you are currently working.  Please circle the number that best fits your level of agreement with each statement, where 5=strongly agree (SA), 4=agree (A), 3=neutral (N), 2=disagree (D), 1=strongly disagree (SD).

| # | **To what extent do you agree with the following statements?** | **SA** | **A** | **N** | **D** | **SD** |
| --- | --- | --- | --- | --- | --- | --- |
| 6 | Considering everything, I am satisfied with my job. | 5 | 4 | 3 | 2 | 1 |
| 7 | Overall, the morale level at my department or section is good. | 5 | 4 | 3 | 2 | 1 |
| 8 | I would encourage my friends and family to seek care here. | 5 | 4 | 3 | 2 | 1 |
| 9 | This is a fun place to work; the work I am doing is stimulating. | 5 | 4 | 3 | 2 | 1 |
| 10 | I am actively involved in helping to make this a great health care facility | 5 | 4 | 3 | 2 | 1 |

**Section III: Working Conditions**

The following questions refer to your working conditions at your current facility.  Encircle the appropriate response.

| # | **To what extent do you agree with the following statement?** | **SA** | **A** | **N** | **D** | **SD** |
| --- | --- | --- | --- | --- | --- | --- |
| 11 | The workload is manageable. | 5 | 4 | 3 | 2 | 1 |
| 12 | I have the supplies I need to do my job well and safely (gloves, needles, bandages, etc). | 5 | 4 | 3 | 2 | 1 |
| 13 | I have the equipment I need to do my job well and efficiently (ultrasound, x-ray, blood pressure cuffs). | 5 | 4 | 3 | 2 | 1 |
| 14 | This facility has good access to drugs and medications. | 5 | 4 | 3 | 2 | 1 |
| 15 | I feel I have job security. | 5 | 4 | 3 | 2 | 1 |

**Section IV: Compensation**

Please indicate your level of agreement with the following questions by marking the appropriate response with a circle.

| # | **To what extent do you agree with the following statements?** | **SA** | **A** | **N** | **D** | **SD** |
| --- | --- | --- | --- | --- | --- | --- |
| 16 | My salary package is fair. | 5 | 4 | 3 | 2 | 1 |

**Section V: Intent to Stay**

Please encircle the most appropriate response to the two questions below.

| **#** | **Questions** | **Encircle your answers** |
| --- | --- | --- |
| 17 | Which of the following statements is true for you? | 1= I would leave this job as soon as possible.  2= I would leave this job within a year from now.  3= I would leave this job one to two years from now.  4= I would leave this job three to five years from now.  5= I plan to stay in this job indefinitely.  6= Other (Specify): _____years |
| 18 | If you want to leave your job soon, which of the following statements best apply to you? | 1= 1 = I would stay with this same organization/employer but would switch to a different location.  2. 2= I would switch to another organization/employer in the Philippines  3= I would switch to a job out of the health sector.  4= I would switch to a job out of the country.  5= I do not want to leave my job soon. |

**Thank you for your cooperation!**

## Appendix B

Point estimates of change from baseline to endline satisfaction scores

|  | **Urban (N=36)** | |  | **Rural (N=54)** | |  | **Remote (N=117)** | |
| --- | --- | --- | --- | --- | --- | --- | --- | --- |
| **Domain** | Change from Baseline | 95% CI †  (L Limit, U.Limit) | | Change from Baseline | 95% CI †  (L. Limit, U.Limit) | | Change from Baseline | 95% CI †  (L. Limit, U.Limit) |
| **Motivation Factors** | |  | |  |  | |  |  |
| Job satisfaction | -0.000029 | (-1.000040, 0.000024) | | -0.000032 | (-0.999951, 0.000021) | | 0.000006 | (-0.000045, 0.999944) |
| Workplace morale | 0.000066 | (-0.999964, 0.500019) | | 0.000030 | (-0.000023, 0.500023) | | 0.999970^**^ | (0.000001, 1.000026) |
| Recommendability | 0.000028 | (-0.999934, 0.999958) | | 0.000031 | (-0.000023, 0.999962) | | 0.499985^**^ | (0.000033, 0.999994) |
| Enjoyment | -0.000064 | (-0.999959, 0.000019) | | 0.000031 | (-0.000019, 0.999965) | | 0.999985^**^ | (0.000023, 1.000074) |
| Job involvement | 0.000019 | (-0.000074, 0.999958) | | 0.000056 | (-0.000012, 0.999984) | | 0.000096 | (-0.000047, 0.999958) |
| **Hygiene Factors** |  |  | |  |  | |  |  |
| Workload | 0.000055 | (-0.000029, 1.000058) | | 0.000037 | (-0.000005, 0.500049) | | 0.000008 | (-0.000025, 0.499971) |
| Access to supplies | 0.999918 | (-0.000014, 1.000000) | | 0.000048 | (-0.499935, 0.500033) | | 0.499936^**^ | (0.000021, 0.999967) |
| Access to equipment | -0.999918 | (-1.000035, 0.000053 | | 0.999930 | (-0.000041, 1.000034) | | -0.000042 | (-0.999967, 0.000027) |
| Access to medicines | 0.000019 | (-0.000074, 0.999958) | | -0.999939^*^ | (-1.000076, -0.000047) | | -0.000020 | (-0.499981, 0.000005) |
| Perceived job security | 0.000049 | (-0.499934, 0.999979) | | 0.000061 | (-0.500000, 0.999941) | | 0.499981^**^ | (0.000047, 0.999957) |
| Compensation | -1.000052**^*^** | (-1.499946, -0.499953) | | -0.999952^*^ | (-1.499983, -0.000062) | | 0.000065 | (-0.000045, 0.499957) |

† based on Hodges-Lehmann estimator of shift in location parameter

* baseline scores < endline scores; increased satisfaction

** baseline score > endline score; decreased satisfaction
